# Ethical imperatives in the commercialization of brain-computer interfaces

**DOI:** 10.1016/j.ibneur.2025.10.004

**Published:** 2025-10-10

**Authors:** Jackson Tyler Boonstra

**Affiliations:** a Faculty of Behavioural and Movement Sciences, Vrije Universiteit Amsterdam, the Netherlands; b Department of Neurology, Amsterdam University Medical Center, the Netherlands

**Keywords:** Brain-computer interfaces, Neuroethics, Data privacy, Regulatory policy

## Abstract

The rapid commercialization of brain-computer interfaces (BCIs) raises urgent ethical and scientific challenges for human research oversight. While BCIs hold transformative potential for treating neurological disorders, their premature translation into consumer markets risks outpacing neuroscientific understanding and ethical frameworks. This essay critically examines the mismatch between commercial claims and the technical limitations of current BCI systems, decoding accuracy and biocompatibility, unresolved ethical dilemmas posed by neural data commodification and procedural risks, and the inadequacy of existing governance to address vulnerabilities in consent, privacy, and long-term safety. Responsible innovation demands proactive measures and robust public engagement to align development with societal values. Without such safeguards, the rush to commercialize BCIs risks prioritizing market interests over patient welfare and eroding public trust in neurotechnology.

## Introduction

The commercialization of brain-computer interfaces (BCIs) has ignited both excitement about transformative medical and cognitive applications and concern over profound scientific, ethical, and social risks. While public demonstrations and media narratives often portray BCIs being on the verge of ‘mind-reading’ or seamless neural control of the newest technology, reality is far more complex. Despite notable advances such as enabling individuals with paralysis to control external devices, BCIs remain largely confined to laboratory and clinical research settings, with limited real-world adoption and persistent technical barriers (Karikari and Koshechkin, 2023a, Peksa and Mamchur, 2023).

Fundamentally, the translation of neural activity into actionable data is constrained by the brain’s distributed, dynamic, and context-sensitive networks, which resist reduction to simple, linear models (Meek et al., 2025, Shen et al., 2023a, Fiani et al., 2021a, Council of Europe, Parliamentary Assembly, 2020). Even the most advanced invasive systems face significant challenges: surgical risks, immune responses, and device degradation over time limit their safety and durability (Maiseli et al., 2023, Shen et al., 2023b). Non-invasive approaches, meanwhile, struggle with low signal resolution and poor robustness, hampering widespread adoption (Karikari and Koshechkin, 2023a, Fried-Oken et al., 2020, Jamil et al., 2021). The scientific community continues to grapple with the fundamental limits of decoding complex brain signal intentions, as well as the need for highly personalized systems that account for individual variability in neural signals and psychological states (Pessoa, 2023, Gallego et al., 2022).

At the same time, the rapid push toward BCI commercialization has outpaced the development of robust ethical, legal, and regulatory frameworks (Chen et al., 2024, Armocida et al., 2024). Issues of data privacy, user autonomy, equitable access, and long-term safety remain unresolved, raising the risk that commercial imperatives overshadow patient welfare and public trust (Almanna et al., 2025, Bublitz, 2019, Naufel and Klein, 2020). This essay critically examines scientific and technical limitations of current BCIs, biological and procedural risks of neural implants, and ethical challenges posed by commodifying neural data and function. Advances are being made towards meaningful public engagement, transparent governance, and new norms for privacy, equity, and autonomy to ensure that neurotechnology evolves in a manner that is both responsible and just.

To enhance the precision of ethical critique, the following working definitions are utilized. *Neural commodification* refers to the process by which a person's uniquely sensitive neural data (*i.e.,* the intimate electrical activity reflecting mental states and identity) is transformed into an economic good (a commodity) to be bought, sold, or leveraged for profit, thereby prioritizing market value over individual autonomy and mental privacy. Correspondingly, *coercive optimism* describes the phenomenon where intense commercial hype and overwhelming promise of transformative medical benefits surrounding neurotechnology unduly influences vulnerable populations (such as patients with severe paralysis) to accept procedural risks or participate in trials, thus undermining truly autonomous and ethically informed consent. *Ethics shopping* is a phrase referring to the practice of companies exploiting variation in regulatory standards and ethical guidelines across different legal jurisdictions in order to minimize compliance burdens (*e.g.,* regarding privacy, animal testing, risk disclosures) by selectively conducting research or trials in locations with the weakest oversight.

## Decoding the brain

The development of BCIs is based on the premise that neural activity can be reliably mapped to specific thoughts or intentions. While early successes in enabling individuals with paralysis to control robotic limbs through imagined movements demonstrate the feasibility of decoding basic motor intentions, these achievements are largely confined to highly controlled experimental settings and simple, repetitive tasks (Dekleva and Collinger, 2025, Dillen et al., 2024). Current BCI systems struggle to generalize beyond these task-specific neural patterns, reflecting a fundamental mismatch between the brain’s dynamic, distributed networks and the discrete, linear models often assumed in BCI design (Kostas and Rudzicz, 2020, Falcon-Caro et al., 2024, Nurse et al., 2015). The intrinsic variability of brain signals, influenced by factors such as attention, fatigue, and individual neuroanatomy, further complicates efforts to achieve robust, real-world decoding of complex intentions (Rué-Queralt et al., 2021, Abiri et al., 2019). Although advanced neuroimaging techniques have shown that certain movement intentions can be predicted from activity, these findings highlight the hierarchical and context-dependent nature of intention coding, rather than a straightforward one-to-one mapping (Meng et al., 2023, Meng et al., 2025). As a result, the field continues to face significant scientific and technical barriers to expanding BCI capabilities beyond basic motor control in controlled environments (Wolpaw et al., 2020, Mridha et al., 2021a, Pang et al., 2024, Saha et al., 2021).

## The illusion of localized intent

Neuroscientific research underscores that even simple actions arise from cascading interactions across multiple brain regions, with neural signals exhibiting plasticity and contextual variability. Studies using neuroimaging and electrophysiology consistently demonstrate that sensorimotor and cognitive tasks are mediated by dynamic network interactions between cortical and subcortical areas, rather than isolated regions (Chumin et al., 2022, Taguchi et al., 2025, Min et al., 2020). For instance, motor cortex activity during hand movement is modulated by environmental cues, emotional states, and prior experience, reflecting both functional and morphological variability in neural activation patterns (Hyde et al., 2017, Xia et al., 2023a, Ejaz et al., 2015). This variability complicates efforts to isolate “pure” intent, as even the same movement can elicit different neural signatures depending on context.

BCIs operate not by reading predefined commands, but by statistically correlating neural patterns with user-generated feedback, a process that requires continuous recalibration and adaptation to individual variability (Ivanov and Chau, 2023, Robinson et al., 2021, Pan et al., 2022). This iterative, feedback-driven approach highlights the brain’s resistance to reductionist interpretations and challenges commercial claims of seamless neural “translation.” Recognizing the distributed and context-sensitive nature of brain activity is essential for realistic expectations of BCI capabilities and limitations (Young et al., 2021, Hughes et al., 2020).

## Dynamic reconstruction

Memory encoding and retrieval present fundamental challenges for BCI development. Unlike static data storage in computers, human memory involves the dynamic recombination of sensory, emotional, and contextual fragments, often influenced by present biases and internal states (McClay et al., 2023, Carpenter and Schacter, 2017, Cox et al., 2023). Research on memory engrams highlights that recalling a single event activates overlapping neural ensembles distributed across the cortex, rather than retrieving a discrete, isolated “file” (Sun et al., 2020, Ghandour et al., 2019, Josselyn and Tonegawa, 2020). This distributed and reconstructive nature of memory means that BCIs cannot simply “read out” memories as digital data.

Attempts to decode semantic content such as distinguishing between memories of different faces or objects currently yield only low-resolution approximations. Some visual BCI studies have managed to reconstruct generalized images or categories from brain activity, but these reconstructions remain coarse and incomplete, more akin to identifying a song from a crowd humming snippets than replaying a precise recording (Josselyn and Tonegawa, 2020, Burke et al., 2015). showing the complexity and limitations in accessing and interpreting nuanced, dynamic neural processes.

This discussion of decoding difficulty must be balanced by acknowledging the notable progress of non-invasive BCI systems utilizing highly robust and unambiguous signals, such as the Steady-State Visual Evoked Potential (SSVEP) (Cheng et al., 2002). SSVEPs are frequency-locked electrical responses in the visual cortex, elicited by targets flickering at a specific rate (Ge et al., 2021)**.** When a user covertly directs spatial attention to a target, the brain's amplified response at that target's unique frequency serves as a direct, decodable signal for communication, even when using shallow scalp EEG probes (Kelly et al., 2005, Müller et al., 1998). This mechanism overcomes some complexities of decoding spontaneous thought by utilizing a straightforward, stimulus-driven neural code to achieve high information transfer rates (Ge et al., 2021)**.** Furthermore, work combining SSVEPs with other paradigms has been used to study the underlying attentional dynamics in visual search, demonstrating how learning can modulate early encephalographic responses to distracting stimuli (Duncan et al., 2025, Forschack et al., 2022). Further technical refinement, such as Rapid Invisible Frequency Tagging (RIFT), utilizes imperceptible flicker rates (e.g., >50 Hz) to maintain the unambiguous BCI signal while minimizing visual fatigue and distraction, thereby demonstrating an exciting pathway for highly efficient, next-generation interfaces (Brickwedde et al., 2022).

## Persistent challenges of neural background activity

BCIs must contend with the brain’s inherent “noise”, or spontaneous neural activity unrelated to user intent that includes subconscious processes, emotional fluctuations, and sensory distractions (Degenhart et al., 2020, Azmi et al., 2023, Vourvopoulos and Bermúdez I Badia, 2016). This background activity can interfere with the detection of goal-directed signals needed for BCI control (Fernández-Rodríguez et al., 2021, Bashford et al., 2018). Even during simple cursor control tasks neural recordings frequently capture competing activity associated with mind-wandering and self-referential thought, diminishing task performance (Martel et al., 2016, May 30, Bradberry et al., 2011, Smallwood et al., 2011, Golub et al., 2016).

Filtering out this neural noise requires not only sophisticated signal processing algorithms but also extensive user training to stabilize and optimize signal patterns (Wei et al., 2020, Piela and Kotas, 2025). Even with advanced methods, achieving consistent accuracy remains a significant challenge, as training can take time and performance often varies due to factors like fatigue and attention (Han et al., 2024, Kwon et al., 2022). Such persistent issues highlight technical and practical barriers to reliable BCI operation in real-world settings.

## Adaptive systems and the limits of generalization

Current BCI systems often require users to adapt their neural activity to the interface, necessitating extensive training so the system can reliably detect intended commands (Vasko et al., 2022, Iwama et al., 2022). However, this user-driven adaptation faces significant scalability challenges: neural patterns trained for a specific task, such as moving a cursor, typically do not generalize well to novel contexts like typing words, leading to the need for repeated and sometimes frustrating recalibration sessions (Saha et al., 2021, Sadtler et al., 2014, Oby et al., 2019). Intra-individual variability including factors such as fatigue, stress, attention, and even age can also significantly degrade BCI performance, highlighting the fragility and instability of current paradigms (Saha and Baumert, 2020, Alimardani and Gherman, 2022, Myrden and Chau, 2015, Zhang et al., 2020). These scientific realities underscore the need for tempered expectations. Experts note framing BCIs as “mind-reading” technologies is misleading; in reality, they are collaborative systems that require mutual adaptation between human and machine, and their effectiveness is highly context-dependent (Rainey et al., 2020, Andorno and Lavazza, 2023, Reardon, 2023). Recognizing this complexity is crucial to avoid hype-driven commodification and ensure realistic progress is made.

## Engineering ambition meets biological reality

Neuralink’s N1 implant, with over a thousand flexible electrodes distributed across ultra-thin threads, marks a significant advance in miniaturization and biocompatibility for BCIs (Fiani et al., 2021a, Shen et al., 2023b, Musk and Neuralink, 2019, Fiani et al., 2021b). However, the brain’s immune response that manifests as glial scarring and chronic inflammation remains a major challenge for all implanted electrodes (Otte et al., 2022, Chae et al., 2024). This biological reaction is well-documented in existing Utah array implants, (Woeppel et al., 2021) which typically maintain high-quality recordings for about two years on average, (Sponheim et al., 2021) though some can last longer, but often experience signal degradation due to tissue response and electrode encapsulation (Cody et al., 2018).

While Neuralink’s polymer-based electrodes are designed to reduce this immune response and minimize implantation damage, there is currently a lack of peer-reviewed, long-term data verifying their durability and sustained performance in humans (Armocida et al., 2024, Musk and Neuralink, 2019, Fiani et al., 2021b, Dadia and Greenbaum, 2019, Drew, 2024). Most published studies on similar flexible polymer electrodes have been short-term and conducted in animal models, leaving Neuralink’s claims of improved longevity and biocompatibility unproven at this stage (Dadia and Greenbaum, 2019, Drew, 2024, Wurth et al., 2017, Krämer, 2024, Naddaf and Drew, 2024). Despite these incremental advances, Neuralink often overpromises by suggesting devices will achieve seamless, long-term integration and high-fidelity neural decoding in humans within a few years, a continuously stated claim that remains unproven. Initial excitement surrounding Neuralink's first human clinical trial, where a quadriplegic man could control a computer cursor with his thoughts, was later tempered by a subsequent setback when a large percentage of the device's threads detached from his brain (Jiang, 2024). While engineering ambitions are driving rapid commercialization of BCIs, confronting complex bioscientific realities is still essential to ensure technologies evolve safely and effectively into real-world applications.

## The ethical weight of device failure

Device malfunction or obsolescence of brain electrodes can necessitate invasive removal surgeries, which expose patients to repeated risks such as hemorrhage, infection, and neural tissue damage (Lozano et al., 2019, Abode-Iyamah et al., 2018, Bjerknes et al., 2014, Tabaja et al., 2022). The risk associated with explanation can be even greater than initial implantation, especially if the device has adhered to tissue or triggered biological responses over time (Leuthardt et al., 2021, Williams et al., 2022). Commercial narratives frequently frame such risks as temporary hurdles rather than systemic limitations, which can obscure the ethical imperative for transparent and comprehensive risk and expectation communication (Council of Europe, Parliamentary Assembly, 2020, Chen et al., 2024, Maynard and Scragg, 2019, McIntosh et al., 2022, Livanis et al., 2024, Burwell et al., 2017). As BCI technology advances under commercial pressures, ensuring that patients are fully informed about both the likelihood and consequences of complications is essential for upholding ethical standards in neurotechnology research and practice (McIntosh et al., 2022, Klein, 2016, Zhang et al., 2024, Versalovic et al., 2020, Padfield et al., 2023).

## Procedural risks in human trials

Invasive BCIs require craniotomies and insertion of electrodes into delicate cortical tissue, with documented procedural risks such as infections, hemorrhages, and potential brain tissue damage (Leuthardt et al., 2021, Mitchell et al., 2023). Clinical studies and regulatory analyses highlight complications including cerebrospinal fluid leaks, seizures, and the possibility of permanent neurological deficits, underscoring the need for rigorous safety protocols and long-term monitoring (Maiseli et al., 2023, Otte et al., 2022, Lozano et al., 2019, Leuthardt et al., 2021, Fenoy and Simpson, 2014, Janssens, 2018, Olson et al., 2023). Animal studies reveal even more serious outcomes. Investigations into Neuralink’s primate research detail cases of fatal complications, such as brain swelling (cerebral edema), chronic infections, partial paralysis, and self-inflicted trauma linked to device-related discomfort, ultimately leading to the euthanasia of several monkeys (Chae et al., 2024, O’Connor, 2023, Physicians Committee for Responsible Medicine, 2024, El País, 2023, Scot Scoop News, 2023). Leaked documents show nearly 1500 animals were killed during the testing of the Neuralink implant; concerns are being raised about the translation of such risks to human trials as commercial pressures mount (Armocida et al., 2024, Drew, 2024, McIntosh et al., 2022, Anand, 2023, Arger, 2023).

Early human trial participants (often individuals with severe disabilities) may view BCIs as their only hope for improved function, making them particularly vulnerable to “coercive optimism” in the informed consent process (Sponheim et al., 2021, Klein, 2016, Gilbert et al., 2017, Cervera et al., 2018). The lack of robust long-term outcome data exacerbates this vulnerability, as the full spectrum of risks and benefits remains uncertain (Maiseli et al., 2023, Robinson et al., 2021, Oby et al., 2019, Sponheim et al., 2021, Wurth et al., 2017). Experts in neuroethics emphasize, when desperation meets unproven technology, there is a real risk that the autonomy of participants becomes compromised by hope and incomplete information (Armocida et al., 2024, Rainey et al., 2020, Dadia and Greenbaum, 2019, Klein, 2016, Adomaitis and Grinbaum, 2024).

## The vulnerability of neural data

BCIs generate continuous streams of neural activity that can reveal deeply personal information, including emotions, intentions, and subconscious processes (Azmi et al., 2023, Paz, 2021, Jazayeri and Ostojic, 2021). Unlike traditional medical records, neural data has the potential to reconstruct internal experiences and even approximate thoughts or visual perceptions, as demonstrated by recent advances in AI decoding of brain signals (Benchetrit et al., 2024). High-resolution interfaces like Neuralink’s raise the stakes further, as they could enable more intrusive inferences, potentially identifying political leanings, mental health states, or other sensitive attributes from neural patterns (Woeppel et al., 2021, Dadia and Greenbaum, 2019, Drew, 2024, Wurth et al., 2017, Krämer, 2024, Naddaf and Drew, 2024, Jiang, 2024). The sheer sensitivity and identifiability of neural data make it uniquely vulnerable to misuse, unauthorized access, and privacy violations (Bublitz, 2019, Naufel and Klein, 2020).

## Commercial exploitation and regulatory void

Existing privacy frameworks such as HIPAA and GDPR were not designed with neural data in mind and often treat it as generic health information, failing to recognize its unique status as a direct window into identity and cognition (American Psychological Association, 2025, Jwa and Poldrack, 2022). The lack of comprehensive, dedicated regulation enables commercial exploitation: companies can collect, store, and even sell neural data to third parties, with data brokers potentially building massive databases of “brain fingerprints” (Jwa and Poldrack, 2022, Yang and Jiang, 2025, Zuk and Lázaro-Muñoz, 2022). This regulatory gap opens the door to dystopian scenarios, including insurers using neural risk factors to deny coverage, employers screening for “undesirable” cognitive traits, and governments surveilling dissent based on brain activity (Council of Europe, Parliamentary Assembly, 2020, Bublitz, 2019, Livanis et al., 2024, Zuk and Lázaro-Muñoz, 2022). Privacy advocates and neuroscientists warn that once neural data leaves an individual’s control, it becomes a highly valuable commodity rarely protected under current legal standards (Paz, 2021, Jwa and Poldrack, 2022, Yang and Jiang, 2025).

## Regulatory gaps and international variation

The FDA classifies most implantable BCIs as Class III devices, requiring rigorous premarket approval focused on safety and efficacy, but current guidance lacks detailed provisions for the ethical handling of neural data and privacy concerns (Council of Europe, Parliamentary Assembly, 2020, Maiseli et al., 2023, Bublitz, 2019, Naufel and Klein, 2020). While the FDA’s regulatory science emphasizes engineering safety and clinical effectiveness, it does not specifically address the unique ethical and privacy risks of neural data processing (Maiseli et al., 2023, Bublitz, 2019, Naufel and Klein, 2020).

In Europe, the Medical Device Regulation (MDR) prioritizes clinical efficacy and safety but provides limited explicit safeguards for neural data privacy. The EU’s AI Act indirectly covers neural data through its regulation of *“emotion recognition systems”* as a subset of biometric data, but this framework is not tailored to the distinct sensitivities of neural information (Council of Europe, Parliamentary Assembly, 2020, Veale and Zuiderveen Borgesius, 2021). China’s regulatory approach is evolving; while the Personal Information Protection Law (PIPL) does not yet clearly define neural data, recent ethical guidelines require mandatory ethical review for BCI research, with a focus on clinical research purposes and state interests (Poo, 2024, Wei et al., 2025). These guidelines emphasize consent and scope of data use but are currently limited in scope and do not fully address data sharing or commercial applications. A global patchwork of standards creates incentives for “ethics shopping,” where companies exploit regulatory weaknesses across jurisdictions to minimize compliance burdens, leaving liability frameworks underdeveloped. The lack of clear legal definitions and tiered liability for neural personal information infringements, illustrates the global challenge of attributing blame to the user, manufacturer, or algorithm designer when adverse outcomes occur (Council of Europe, Parliamentary Assembly, 2020, Naufel and Klein, 2020).

## Building trust through engagement

A nationwide survey found that the majority of Americans consider brain data as sensitive as, or even more sensitive than, genetic or financial data, and many are worried about its misuse by corporations and other entities (Almanna et al., 2025, Jiang, 2024). Researchers themselves report concerns about the potential harms and risks of misuse if neural data were to be shared or exploited, especially for vulnerable populations (Leuthardt et al., 2021, Klein, 2016, Jwa and Poldrack, 2022). Restoring confidence demands inclusive oversight: neuroethics committees and governance frameworks should integrate patients, ethicists, and civil society representatives to co-design policy and oversight mechanisms.

Recent neuroethics guidance documents emphasize the importance of multi-stakeholder engagement-including patients, ethicists, and community representatives-in developing responsible governance for neurotechnology and brain data (Armocida et al., 2024, Rainey et al., 2020, Dadia and Greenbaum, 2019, Maynard and Scragg, 2019, McIntosh et al., 2022, Livanis et al., 2024, Burwell et al., 2017, Klein, 2016, Physicians Committee for Responsible Medicine, 2024). Experts and policy reports argue that transparency, public engagement, and open-source approaches are essential for building trust and aligning neurotechnology development with societal values (Goering et al., 2021, Güemes et al., 2024). Neuroethics literature further recommends independent monitoring, public reporting of risks, and temporary bans on enhancements lacking clear therapeutic justification as safeguards to protect public trust and prevent ethical lapses (Council of Europe, Parliamentary Assembly, 2020, Bublitz, 2019, Naufel and Klein, 2020, American Psychological Association, 2025, Yang and Jiang, 2025).

To move beyond abstract calls for multi-stakeholder engagement, governance of neural data and BCIs must be grounded in specialized, concrete frameworks. First, legal frameworks need to explicitly recognize the unique sensitivity of neural data, since existing privacy laws were not designed to address information capable of revealing mental states (Ienca et al., 2021, Xia et al., 2023b). Recent legislative reforms in U.S. states such as Colorado and California have begun incorporating neural data into privacy laws, representing an important but incomplete step toward robust protections (Mridha et al., 2021b). Second, oversight models should be formalized beyond the limited scope of traditional Institutional Review Boards (IRBs), that lack agility in addressing consumer, workplace, and military applications of BCI (Naufel and Klein, 2019, Wahlstrom et al., 2017). Independent, multi-stakeholder, and engineering-focused frameworks, such as the IEEE Neuroethics Framework, provide structured guidance for evaluating the ethical, legal, and sociocultural implications (ELSCI) of neurotechnologies (Sample et al., 2019). Finally, regulatory models should distinguish between therapeutic and augmentative BCIs, with several scholars urging moderation in augmentative applications to prevent exacerbating social inequalities (Karikari and Koshechkin, 2023b, Edelman et al., 2024).

## Conclusion

BCI technology stands at a crossroads: it could redefine human potential or deepen existing vulnerabilities. The path forward requires humility in recognizing our understanding of the brain remains rudimentary, therefore we must be vigilant against commodification masquerading as fundamental progress. The brain is not a frontier to be conquered–but a mystery to be approached with reverence. Only through rigorous science, thoughtful ethics, and unwavering accountability can neurotechnology honor this complexity.

## Compliance with Ethical Standards

This review article does not involve original human or animal research conducted by the author. It is based solely on the analysis and synthesis of existing literature and publicly available data. All sources are appropriately cited, and the work adheres to the ethical standards of academic publishing, including those outlined by IBRO Neuroscience Reports and Elsevier. No ethical approval was required for this study.

## Declaration of Competing Interest

I, Jackson Tyler Boonstra, PhD, declare no conflicts of interest related to the submitted review article, “Ethical Imperatives in the Commercialization of Brain-Computer Interfaces.” I have no financial, personal, or professional relationships with individuals or organizations that could influence the work presented in this manuscript.
